# Genetic approach to track neural cell fate decisions using human embryonic stem cells

**DOI:** 10.1007/s13238-013-0007-y

**Published:** 2014-01-29

**Authors:** Xuemei Fu, Zhili Rong, Shengyun Zhu, Xiaocheng Wang, Yang Xu, Blue B. Lake

**Affiliations:** 1 Shenzhen Children’s Hospital, Shenzhen, 518026 China; 2 Section of Molecular Biology, Division of Biological Sciences, University of California, San Diego, 9500 Gilman Drive, La Jolla, CA 92093-0322 USA

**Keywords:** Nestin, knock-in, human embryonic stem cells, neural progenitor cells

## Abstract

With their capability to undergo unlimited self-renewal and to differentiate into all cell types in the body, human embryonic stem cells (hESCs) hold great promise in human cell therapy. However, there are limited tools for easily identifying and isolating live hESC-derived cells. To track hESC-derived neural progenitor cells (NPCs), we applied homologous recombination to knock-in the *mCherry* gene into the *Nestin* locus of hESCs. This facilitated the genetic labeling of Nestin positive neural progenitor cells with mCherry. Our reporter system enables the visualization of neural induction from hESCs both *in vitro* (embryoid bodies) and *in vivo* (teratomas). This system also permits the identification of different neural subpopulations based on the intensity of our fluorescent reporter. In this context, a high level of mCherry expression showed enrichment for neural progenitors, while lower mCherry corresponded with more committed neural states. Combination of mCherry high expression with cell surface antigen staining enabled further enrichment of hESC-derived NPCs. These mCherry^+^ NPCs could be expanded in culture and their differentiation resulted in a down-regulation of mCherry consistent with the loss of Nestin expression. Therefore, we have developed a fluorescent reporter system that can be used to trace neural differentiation events of hESCs.

## Introduction

Human embryonic stem cells (hESCs) undergo unlimited self-renewal and retain pluripotency to differentiate into all cell types of the body, making them invaluable tools for development of cellular therapeutics (Fu and Xu, 2011). Modeling human disease has further become possible with advances in genetic modification in hESCs either through homologous recombination (Song et al., 2010; Hockemeyer et al., 2011) or gene knockdown (Ordonez et al., 2012), as well as reprogramming of cells having disease-causing mutations into an ESC-like state (human induced pluripotent stem cells or hiPSCs) (Unternaehrer and Daley, 2011). Given the limitation of using mouse systems to develop human therapeutics, the use of these approaches can provide more physiologically relevant insight into disease pathology and help to identify more efficacious drug targets. These approaches additionally permit disease modeling of tissues that are previously unattainable or that show pathologies too advanced in donor tissues. This includes studies on the mechanisms underlying neuronal degeneration associated with central nervous system (CNS) diseases (e.g. Parkinson’s, Alzheimer’s or Amyotrophic lateral sclerosis).

Studies using human pluripotent stem cell derived neurons to model these diseases or generate neural tissue replacements rely upon the effectiveness of protocols that recapitulate normal embryonic development to direct the differentiation of hESCs into the neural cell types afflicted by the disease (Aas et al., 1996). A key step in this process is to differentiate hESCs into neural stem cells (NSCs) that can be further differentiated into neurons, astrocytes and glia. While significant progress has been made in improving the differentiation of hESCs into NSCs (Reubinoff et al., 2001; Itsykson et al., 2005; Wu et al., 2007; Chambers et al., 2009), this step remains an imperfect process with resulting cultures comprising a mixture of undifferentiated ESCs, NSCs, neurons, glia and non-CNS cell types including neural crest. Subsequent differentiation of these mixed cultures would fail to generate single pure lineages needed for transplantation, modeling or population based gene expression studies (e.g. RNA-seq).

To circumvent these issues, several groups have had success in using combinatorial cell surface marker staining to enrich for NSCs (Yuan et al., 2011; Israel et al., 2012). However this method requires further sorting strategies for continued monitoring of resultant cell differentiation states. Promoter-based viral reporter systems for the neural progenitor marker *Nestin* has also been used to specifically label NSCs within the human fetal brain and could be used to purify these cells for expansion or for further differentiation both in culture and following transplantation (Keyoung et al., 2001). Several groups have used *Nestin* reporter systems to identify and track neural progenitors both *in vivo* and *in vitro* (Sawamoto et al., 2001; Lenka et al., 2002; Mignone et al., 2004; Noisa et al., 2010). These transgenic or viral based strategies, however, involve integration of the exogenous DNA into random genomic loci that can modulate the promoter activity, depending on the epigenetic state around the integration site. To address this issue, we decided to generate knock-in cell lines using a *Nestin*-based fluorescent reporter system that would permit not only a simpler sorting strategy for isolating NPCs/NSCs but also would enable their live tracking during expansion or differentiation. Unlike transgenic approaches (Placantonakis et al., 2009), this system would ensure reporter expression specifically driven by the endogenous *Nestin* promoter and/or enhancer, and would not introduce any other genetic alterations that arise from random integration strategies. This would permit specific mCherry expression during neural differentiation of hESCs and the ability to generate enriched NSC cultures where multi-potentiality could be easily monitored. These cells will provide a unique opportunity for studies requiring purer neural cultures and live cell tracking, including transplantation, disease modeling or whole transcriptome analyses (Peljto and Wichterle, 2011).

## Results

### Generation of a knock-in Nestin reporter cell line

**Figure 1:**
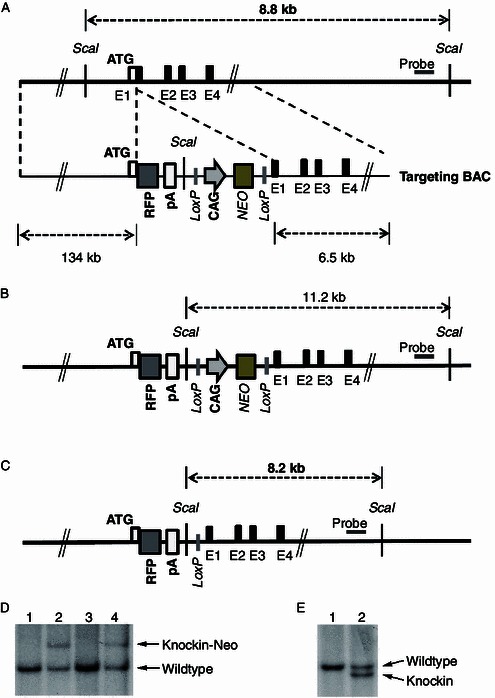
*Nestin:mCherry* knock-in strategy. (A) The germline configuration of the *Nestin* locus in hESCs showing: the exon containing the initiation ATG; the germline ScaI fragment; the probe used for Southern blot analysis; and the BAC targeting vector containing the *mCherry* (RFP) expression cassette together with the LoxP-flanked selection marker. (B) Insertion of this cassette into the *Nestin* exon replaced the initiation ATG of *mCherry* with that of the *Nestin* gene and generated an altered ScaI fragment size corresponding to the targeted allele. (C) The transient expression of Cre led to the excision of the selection marker allowing normal expression of the *mCherry* according to the endogenous *Nestin* gene. (D) Southern blot analysis of the targeted hESCs. Genomic DNA was digested with ScaI and hybridized to the probe indicated in (A and B). The location of the targeted and wild-type fragments are indicated. Lanes 1 and 3: hESCs with random integration of the targeting construct. Lanes 2 and 4: homologous recombinants. (E) Southern blot analysis of the knock-in hESCs showing that the selection marker was excised from the targeted allele by LoxP/Cre-mediated deletion. Genomic DNA was digested with ScaI and probed with the same targeting probe shown in (A, C)

**Figure 2:**
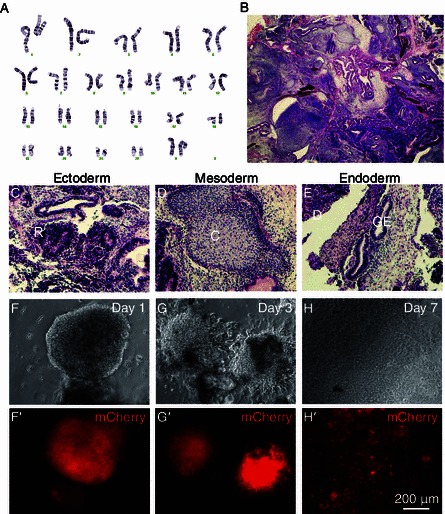
*Nestin-mCherry* knock-in hESCs. (A) Twenty metaphase cells of the knock-in hESCs were examined and exhibited normal karyotypes. (B) Knock-in hESCs formed well-differentiated teratomas in SCID mice. Cells of each of the three germ layers were identified in the teratomas including: (C) ectoderm (R, neural rosette); (D) mesoderm (C, cartilage); (E) endoderm (GE, gut-like epithelium). Out migration of mCherry positive neural cells were observed from teratomas formed by knock-in hESCs on day 1 (F–F′), day 3 (G–G′) and day 7 (H–H′) following attachment in NPC medium

We have recently developed a bacterial artificial chromosome (BAC) based targeting strategy that enables a high efficiency of homologous recombination in hESCs (Song et al., 2010). To knock-in the *mCherry* coding region into the *Nestin* locus, we constructed a BAC targeting vector in which the ATG of one endogenous *Nestin* allele was replaced with that of *mCherry* (Fig. 1A and 1B). In order to screen for homologous recombinants, one homologous arm of the BAC vector was shortened to 6.5 kb. Recombinants were then screened by Southern blotting to identify both the 8.8 kb germline band and the 11.2 kb mutant band (Fig. 1B). The LoxP-flanked selection marker cassette (*CAG-Neo*) was subsequently excised from the genome (Fig. 1C) through transient expression of the Cre enzyme (Song et al., 2007). Knock-in hESCs showed normal karyotypes (Fig. 2A) and retained the potential to differentiate into all three germ layers including neuroectoderm (Fig. 2B and 2C). Furthermore, mCherry-positive neural-like cells were found to migrate from teratoma tissues when they were cultured in neural progenitor medium (Fig. 2F–H). Therefore, our knock-in cell line may provide a means for monitoring the formation of neural lineages during early hESC differentiation.

### mCherry fluorescence follows Nestin expression dynamics

**Figure 3:**
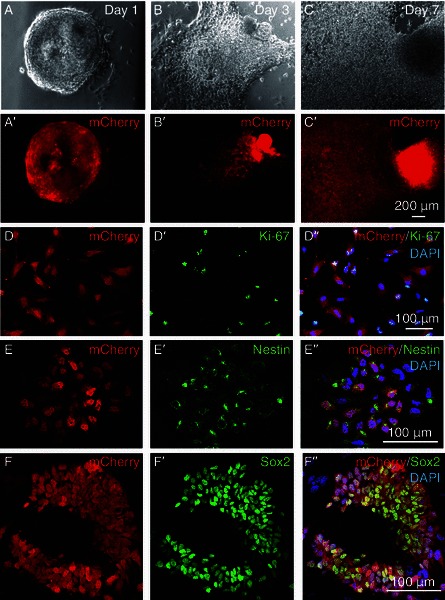
mCherry labeled Nestin positive neural progenitors derived from knock-in hESCs. Neural induction of embryoid bodies (EB) and corresponding mCherry expression on day 1 (A–A′), day 3 (B–B′) and day 7 (C–C′) following EB attachment. mCherry positive neural progenitor cells cultured in NPC medium showed active proliferation as indicated by Ki-67 staining (D–D′′), co-expressed Nestin (E–E′′) and co-localized with Sox2 in rosette-like structures (F–F′′)

To further validate whether our genetic tagging of the *Nestin* locus could allow tracing of neural differentiation from hESCs, *Nestin:mCherry* hESCs were differentiated with an established embryoid body (EB) neural induction protocol (Reubinoff et al., 2001; Zhang et al., 2001). mCherry positive cells could be observed soon after EB treatment with neural induction medium coincident with the expected emergence of neural progenitors (Fig. 3A). Further, mCherry positive cells were found to migrate out from attached EBs (Fig. 3B and 3C) and rapidly expanded to confluence similarly to that observed for teratoma tissues (Fig. 2F–H). This confirms that our *Nestin:mCherry* reporter system can monitor neural specification from both *in vitro* and *in vivo* differentiated hESCs.

Following out-migration from EB or teratoma tissues, mCherry positive cells displayed properties consistent with neural progenitor cells. They could be further expanded in culture for several passages (data not shown); showed significant proliferation as indicated by Ki-67 staining (Fig. 3D); and co-expressed endogenous Nestin (Fig. 3E). Furthermore, high mCherry expression was localized to neural rosette-like structures, as was previously reported for a transgenic *Nestin* reporter (Noisa et al., 2010), and co-localized with the neural stem cell marker Sox2 (Fig. 3F). Therefore, mCherry expression can faithfully label Nestin positive cycling progenitors during differentiation of hESCs into the neural lineage both *in vitro* and *in vivo*.

### mCherry levels dropped during NPC differentiation

**Figure 4:**
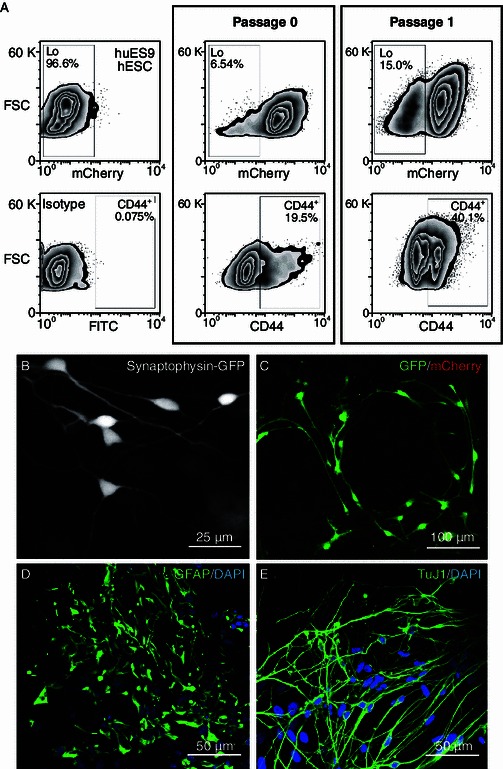
mCherry intensity is consistent with *Nestin* expression dynamics. (A) FACS analysis for mCherry expression levels in Hues9 hESCs or neural cultures derived from *Nestin:mCherry* knock-in Hues9 hESCs. Neural cultures that were out-migrating from EBs (Passage 0) or were passaged one time (Passage 1) were either stained with FITC isotype control or FITC labeled anti-CD44 antibody. Both mCherry low expressing fractions and CD44 positive fractions are indicated. (B) mCherry^Hi^ population differentiates into synapsin positive neurons as shown by *Syn:GFP* reporter lentivirus. (C) mCherry expression is lost in terminally differentiated neurons identified by the *Syn:GFP* reporter. mCherry^Hi^ NPCs can be differentiated into GFAP^+^ astrocytes (D) and Tuj1^+^ neurons (E)

**Figure 5:**
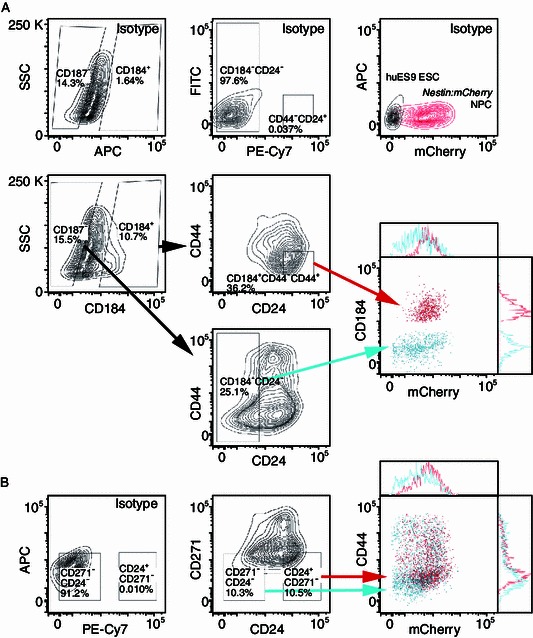
NSC markers map to the mCherry^Hi^ Population. *Nestin:mCherry* NPCs at P0 were stained with isotype control antibodies or FITC labeled anti-CD44, PE-Cy7 labeled anti-CD24, Alexa-647 anti-CD271 or APC labeled anti-CD184 antibodies. huES9 ES cells were used as an mCherry negative control. (A) CD184^+^ cells were gated on CD44^-^CD24^+^, while CD184^-^ were gated on CD24^-^ and both populations were shown relative to the CD184 and mCherry distributions. (B) CD271^-^CD24^-^ and CD271^-^CD24^+^ populations were shown with respect to CD44 and mCherry distributions

While our results indicate that the knock-in of *mCherry* into the *Nestin* locus recapitulates its endogenous expression, we next sought to define whether mCherry specifically labels neural progenitors. To do address this, mCherry positive cells were characterized using flow cytometry (Figs. 4A and 5). Interestingly, two different fractions showing low and high mCherry expression were revealed with global passaging of neural cultures coincident with an emerging population of CD44 positive cells (Fig. 4A) representing more differentiated neural cell types (Liu et al., 2004). Continued culture in this manner resulted in the progressive loss of mCherry expression over several passages (data not shown). Our results are consistent with mCherry^Lo^ cells potentially representing more committed neural cell types as has been previously reported for a transgenic hESC system (Noisa et al., 2010). In fact, mouse Nestin reporters exhibited gradient levels of reporter activity during differentiation, with high levels associated with neural stem cells, lower levels observed in more differentiated yet dividing neuronal progenitors and finally little to no activity in fully differentiated neurons (Sawamoto et al., 2001; Lenka et al., 2002; Mignone et al., 2004). Consistently, mCherry expression was lost in terminally differentiated neurons that were labeled with a synapsin-GFP lentiviral reporter (Fig. 4B and 4C). In addition, mCherry^Hi^ NPCs could be differentiated into both GFAP^+^ astrocytes and Tuj1^+^ neurons (Fig. 4D and 4E), confirming their multipotency. Therefore, our results demonstrate that mCherry levels correspond with endogenous *Nestin* expression, specifically labeling neural progenitors and lost upon their further differentiation.

One significant advantage to the labeling of NPCs with mCherry would be the potential for sorting and enriching this population. Furthermore, given the potential heterogeneity of neural progenitors in terms of stage of commitment and potentiality, it would be advantageous to specifically isolate or enrich bone fide neural stem cells. To determine the specificity of mCherry to NSCs or NPCs compared to more committed cell lineages, we used cell surface markers that highlight these different neural subpopulations. Previous reports have shown the use of multiple surface markers to identify NSCs based on a CD184^+^CD271^-^CD44^-^CD24^+^ staining signature (Yuan et al., 2011). Both CD271 and CD44 identify undifferentiated hESCs, as well as neural contaminants including neural crest stem cells, peripheral neural cells, more differentiated neuronal cell types, astrocyte progenitors and glia (Morrison et al., 1999; Luo et al., 2002; Liu et al., 2004; Lee et al., 2007). Alternatively, both CD184 and CD24 have been shown to be expressed on the surface of NSCs. Consistent with mCherry high expressers representing a more neural stem cell state, the CD184^+^CD44^-^CD24^+^ population localized more within the mCherry^Hi^ expressing cells in contrast to the CD184^-^CD24^-^ population (Fig. 5). Furthermore, the CD271^-^CD44^-^CD24^+^ cells also mapped more to the mCherry^Hi^ expressers unlike a similar CD24^-^ population (Fig. 5). These results indicate that mCherry high expression is in part predicative of a stem cell state, but is not specific to this population (Fig. 5). This is likely the result of continued high Nestin expression in more committed neural progenitors as well as a potential lag in the mCherry expression compared to endogenous Nestin that is a result of their differing protein stabilities.

### mCherry sorting strategy for NSC enrichment

**Figure 6:**
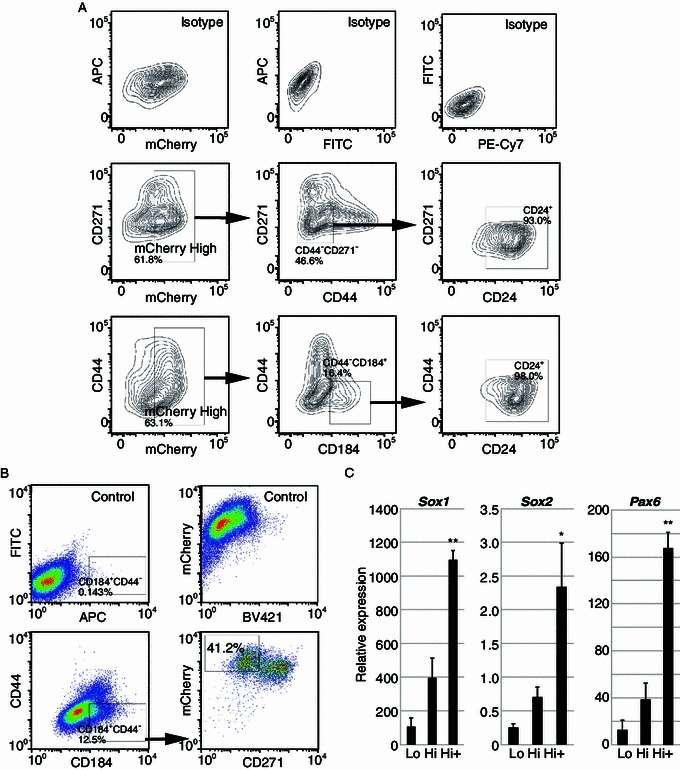
mCherry^Hi^-based sorting strategy significantly enriches NSCs. (A) *Nestin:mCherry* NPCs at P0 were stained with isotype control antibodies or FITC labeled anti-CD44, PE-Cy7 labeled anti-CD24, Alexa-647 anti-CD271 or APC labeled anti-CD184 antibodies. mCherry^Hi^ cells were gated on CD271^-^CD44^-^ or CD44^-^CD184^+^ and shown with respect to CD44 and CD24 distributions. (B) *Nestin:mCherry* NPCs at P0 were either unstained (Control) or stained with FITC labeled anti-CD44, BV421 labeled anti-CD271 and APC labeled anti-CD184 antibodies. The CD184^+^CD44^-^ cells (all gating based on isotype control staining) were shown with respect to mCherry and CD271 distributions. The mCherry^Hi^CD271^-^ population was sorted for gene expression analyses. (C) mCherry^Lo^ (Lo), mCherry^Hi^ (Hi), mCherry^Hi^ CD184^+^CD271^-^CD44^-^ (Hi +) populations were sorted from P0 *Nestin:mCherry* NPCs and real time PCR analysis was performed for *Sox1, Sox2* and *Pax6* expression relative to undifferentiated *Nestin:mCherry* huES9 hESCs. Results shown are from two independent experiments. *P < 0.05 and **P < 0.01 (two-tailed *T*-test relative to the mCherry^Lo^ condition)

While high mCherry expression occurs in neural stem cells (Fig. 3F), it in itself is not sufficient to fully purify this fraction from the global population. In order to achieve this, mCherry^Hi^ expression would need to be combined with cell surface marker staining. Interestingly, neural cultures that were gated on the mCherry^Hi^ expression and that were additionally either CD271^-^CD44^-^ or CD44^-^CD184^+^ (Fig. 6A) were almost entirely CD24^+^ when neural cells were collected directly from induced EBs. This indicates a redundancy for this marker at this stage in neural differentiation. However, this trend was not necessarily observed after passaging of the total population, likely due to the spontaneous differentiation of NPCs (data not shown). Therefore, these results indicate that mCherry^Hi^ can substitute for the CD24^+^ staining when isolating NSCs directly from induced EBs, enabling a simpler sorting strategy for these cells. Consistently, the sorted CD184^+^CD44^-^CD271^-^ population, which was found to map to a mCherry^Hi^ population (Fig. 6B), showed significant further enrichment of the NSC markers *Sox1*, *Sox2* and *Pax6* over the mCherry^Hi^ population alone (Fig. 6C). Use of dual SMAD inhibitors Noggin and SB431542 during neural induction (Chambers et al., 2009) was found to significantly increase the number of NSCs sorted and showed a further increase in *Sox1* and *Pax6* expression levels in the enriched mCherry^Hi^ population (data not shown). Therefore, these results indicate that the combination of mCherry^Hi^ with a more limited surface marker sorting strategy that excludes CD24 can be sufficient to significantly enrich for NSCs. Furthermore, substituting CD24 for mCherry expression provides the distinct advantage of being able to further monitor the differentiation status of the NPC culture and permits tracking of terminal differentiation events.

## Discussion

Our studies demonstrate a knock-in approach that can be used together with existing strategies of purifying NPCs/NSCs derived from hESCs while additionally providing a means of tracking these cells live, both *in vitro* and *in vivo*. Transgenic or viral *Nestin* reporters have been widely used to label neural progenitors (Keyoung et al., 2001; Sawamoto et al., 2001; Lenka et al., 2002; Mignone et al., 2004; Noisa et al., 2010) and Nestin positivity remains an important marker identifying hESC-derived NSCs (Reubinoff et al., 2001; Zhang et al., 2001; Itsykson et al., 2005; Joannides et al., 2007; Chambers et al., 2009). Our transgene-free *Nestin:mCherry* reporter hESC line provides the unique ability to monitor the differentiation status of our cultures with the sensitivity to distinguish high and low sub-populations of Nestin expression and without requiring Nestin immunostaining. While mCherry^Lo^ cells likely correspond to more committed neural lineages or non-neural contaminants, the mCherry^Hi^ population appears to enrich for neural progenitors with multi-lineage capabilities. Furthermore, the combination of mCherry^Hi^ with CD184, CD44 and CD271 surface marker staining permitted significant enrichment of NSC-specific markers (*Sox1*, *Sox2* and *Pax6*). These results indicate that our reporter system can be combined with additional strategies for specifically isolating or enriching NSCs including and not limited to: surface marker staining; additional knock-in strategies conferring NSC or lineage-specific drug resistance; or knock-in fluorescent reporters of additional neural lineage markers. Furthermore, our knock-in line was able to achieve significant enrichment of NSCs with a smaller subset of surface markers (CD184^+^CD44^-^CD271^-^) as has been previously used (Yuan et al., 2011), providing a clear advantage over wild-type hESCs. The potential to replace the CD24 surface marker with intrinsic fluorescent reporter activity further provides a means to monitor the NSC cultures and track terminal differentiation. Our lineage-specific knock-in approach will enable further studies tracing the commitment of neural progenitors derived from hESCs; to perform population based studies; and to improve current protocols for directed differentiation of cell types needed for cell replacement therapy.

## Materials and methods

### Construction of BAC-based targeting vector

The *Nestin* BAC clone was purchased from Life Technologies Inc. and the targeting vector was constructed by recombineering as we previously described (Song et al., 2010).

### Cell culture of hESCs

The HUES9 hESCs were cultured as described (Cowan et al., 2004). Briefly, hESCs were cultured on mouse embryonic fibroblast (MEFs) feeder layer in knockout medium supplemented with 10% knockout serum replacement, 10% plasmanate, 1 mmol/L glutamine, 0.1 mmol/L nonessential amino acids, 10 ng/mL bFGF, and 0.1 μmol/L β-mercaptoethanol. To passage hESCs, confluent culture was washed with phosphate buffered saline (PBS), dissociated for 5 min with TrypLE and resuspended into single cells with culture medium. All tissue culture reagents were purchased from Life Technologies Inc. except where indicated otherwise.

### Differentiation of hESCs into neural progenitors

The neural differentiation of hESCs was performed with established protocols (Reubinoff et al., 2001; Zhang et al., 2001). Briefly, hESC colonies were gently scraped from the plate and cultured in suspension as embryoid bodies (EB) on ultra-low cluster plates in EB formation medium (DMEM/F12-Glutamax, 10% characterized FBS, 1% Pen-Strep, 100 μmol/L β-mercaptoethanol). The media was changed to NPC induction medium (NPC medium: DMEM/F12-Glutamax, 0.5% N2, 1% B27, 1% Penicillin-Streptomycin, 10 ng/mL bFGF). After two days, the EBs were replated onto poly-L-ornithine/laminin coated plates. After neural cells migrated away from the EBs and proliferated to confluence, the culture was treated with 0.05% trypsin for 1–2 min at room temperature, resuspended into single cells, and either sorted or re-plated onto poly-L-ornithine/laminin coated dishes in NPC medium. In one sort experiment, BMP inhibitor Noggin (500 ng/mL, R&D Systems) and TGF-beta inhibitor SB431542 (10 μmol/L, Sigma-Aldrich) were included during EB differentiation stages prior to their attachment onto poly-L-ornithine/laminin coated dishes.

### Teratoma formation of hESCs in SCID mice

Two million hESCs were suspended in the knockout medium supplemented with 30% matrigel (BD Biosciences) and injected subcutaneously into severe combined immunodeficiency (SCID) mice. After six to eight weeks, teratomas were recovered. For histological assessment, teratomas were fixed in 10% buffered formalin, embedded in paraffin, sectioned and stained with hematoxylin and eosin. To derive NPCs from teratomas, teratomas were chopped into small pieces and cultured in NPC medium. After several passages, the adherent culture became homogenous with mCherry positive cells.

### Differentiation of hESC-derived NPCs into mature neurons

The hESC-derived neural progenitors were transduced with lentivirus harboring synapsin promoter-driven EGFP as described (Kim et al., 2011; Lake et al., 2012). The NPCs were cultured in the neuronal differentiation medium containing DMEM/F12 supplemented with N2/B27, BDNF (10 ng/mL, Peprotech), GDNF (10 ng/mL, Peprotech), Penicillin-Streptomycin (Invitrogen), glutamax (Invitrogen), cyclic AMP (1 μmol/L, Sigma Aldrich). The cells were cultured in differentiation media for two to three weeks with 50% of the medium replaced every other day.

### Quantitative real-time PCR analysis

Total RNA from hESCs and hESC-derived neural cultures were isolated using RNAeasy Mini Kit (QIAGEN) or ZR RNA MicroPrep Kit (Zymo Research). Total RNA was reverse transcribed using the High Capacity cDNA Reverse Transcription Kit (Applied Biosystems) and analyzed on a StepOnePlus™ quantitative real-time PCR system (Applied Biosystems) as previously described (Song et al., 2007). The primers used are as following: *Sox1* (F-GAGTGGAAGGTCATGTCCGAGG; R-CCTTCTTGAGCAGCGTCTTGGT), *Sox2* (F-ATGCACCGCTACGACGTGA; R-CTTTTGCACCCCTCCCATTT), *Pax6* (F-TGTCCAACGGATGTGTGAGT; R-TTTCCCAAGCAAAGATGGAC), *GAPDH* (F-GATGACATCAAGAAGGTGGTGA; R-GTCTACATGGCAACTGTGAGGA). Data analysis was performed using Microsoft Excel.

### Immunofluorescence staining

Neural progenitor cells grown on poly-L-ornithine/laminin coated 4-well chamber plates were fixed in 4% paraformaldehyde for 15 min at room temperature, permeabilized with 0.3% Triton-X-100 in Triton Buffer Solution (TBS) for 10 min, blocked with 2% FBS in TBS-0.05% Tween-20 for 20 min and stained with primary antibodies in blocking buffer at 4°C overnight. Primary antibodies included: anti-Nestin (611658; BD Pharmingen); anti-SOX2 (561469; BD Pharmingen); anti-RFP (600-401-379; Rockland); anti-ki67-Alexa fluor-488 (51-9007231; BD Stemflow Human neural Lineage analysis Kit); TuJ1 (MMS-435P, Covance); GFAP (ab7779, Abcam). The next day, the cells were stained with secondary antibodies (Alexa fluor 568 donkey anti-rabbit; Alexa fluor 488 donkey anti-mouse; Life Technologies, Inc.) in blocking buffer for 45 min and nuclei were counterstained with DAPI. Images were acquired using an Olympus FV1000 confocal microscope. Live culture images were also acquired using an Inverted Axioscope and AxioCam MRm (Carl Zeiss, Inc.). Image assembly was performed using Adobe Photoshop CS5 (Adobe Systems, Inc.).

### Flow cytometry

hESC-derived neural cultures were washed with PBS, trypsinized with 0.05% trypsin for 1–2 min at room temperature and resuspended into single cells using DMEM/F12 with Glutamax containing 10% FBS. For cell surface staining, cells were incubated with CD184-APC (560936; BD Pharmingen), CD24-PE-Cy7 (561646; BD Pharmingen), CD44-PE (51-9007231; BD Pharmingen) and/or CD271-Alexa fluor 647 or BV421 (560877 or 562562; BD Pharmingen) antibodies in PBS containing 1% bovine serum albumen (BSA) according to manufacturer’s protocol. Stained and unstained cells were analyzed using either a BD LSR-II or LSRFortessa flow cytometry machine (BD Biosciences). Control hES9 hESCs or hESC-derived NPCs were used for mCherry control and IgG-PE-Cy7, IgM-FITC, IgG-BV421 and IgG-APC (BD Pharmingen) were used for isotype controls. Cell sorting was performed using either BD FACSAria II or BD Influx cell sorter. Gating for all sorts was defined by isotype control staining. Flow cytometry data analysis was performed using FlowJo software (Tree Star, Inc.).
